# Lesion conspicuity by size in [^18^F]FDG long-axial field-of-view PET/CT

**DOI:** 10.1186/s13550-026-01374-3

**Published:** 2026-01-13

**Authors:** Thomas Pyka, Luis Weissenrieder, Konstantinos Zeimpekis, Hasan Sari, Federico Caobelli, Kevin J. Chung, Lorenzo Nardo, Axel Rominger, Clemens Mingels

**Affiliations:** 1 Department of Nuclear Medicine Inselspital, Bern University Hospital, University of Bern, Freiburgstr. 18, Bern, 3010 Switzerland; 2 TUM School of Medicine and Health, Munich, Germany; 3 Department of Radiology, University of California Davis, Sacramento, CA USA; 4 Advanced Clinical Imaging Technology, Siemens Healthcare AG, Lausanne, Switzerland

## Introduction

The introduction of long-axial field-of-view (LAFOV) and Total-Body (TB) positron emission tomography/computed tomography (PET/CT) scanners has changed the field of molecular imaging [1–4]. Compared to conventional short-axial field-of-view (SAFOV) systems, digital LAFOV PET shows increased sensitivity, positron yield and spatial resolution [5–7]. This results in a significant reduction of injected activities and scan time [8, 9].

[^18^F]FDG PET/CT is an established tool for staging, restaging, treatment monitoring and response assessment in a variety of oncologic diseases, which is reflected by multiple of guidelines (e.g. in lymphoma, non-small cell lung cancer (NSCLC), melanoma etc.) [10, 11]. Proper lesion quantification is crucial for its interpretation. Several scoring systems and therapy assessment tools (e.g. like the Deauville and Lugano classification) rely on comparing lesion [^18^F]FDG uptake to a reference organ like the liver [12]. Lesion quantification might differ for each PET system owing to individual reconstruction algorithms and different scanner characteristics [9]. In addition, accurate PET-quantification in small lesions is particularly difficult because of the partial volume effect [13].

However, LAFOV PET-systems have shown to be able to properly quantify small lesions with low uptake and in unfavorable conditions like increased blood glucose levels [14, 15]. Detection of small or low FDG-avid lesions can change the therapeutical regimes drastically [16]. We therefore aimed to compare lesion quantifications on LAFOV and simulated SAFOV (sSAFOV) PET/CT and determine new image quality criteria, which take its increased sensitivity into account. We hypothesized that on LAFOV PET/CT small lesions show superior tracer uptake with LAFOV compared to sSAFOV PET/CT. The secondary outcome was to assess the image quality per lesion size and compare small lesions to larger ones on LAFOV and sSAFOV.

## Materials and methods

### Patient population and imaging

57 oncologic patients undergoing clinical routine [^18^F]FDG LAFOV PET/CT were retrospectively evaluated. Patients fasted for at least 6 h prior to the PET/CT and blood glucose levels were according to the EANM guidelines < 11.0mmol/l [17]. In total, 3.0±0.19 MBq/kg [^18^F]FDG was injected intravenously. Patients received skull base to mid thighs PET on the Biograph Vision Quadra (Siemens Healthineers, Erlangen, Germany) LAFOV PET/CT. A 360 s list-mode scan was acquired in maximum ring difference 322 (MRD 322, UHS). To simulate SAFOV PET images, PET data were reconstructed in MRD 85 (HS) and 120 s acquisition time. Image reconstruction was performed using the vendor’s standard reconstruction software (e7-tools, Siemens Healthineers), with OSEM (4 iterations, 5 subsets), point-spread-function (PSF), and TOF enabled as previously published [15]. Images were reconstructed with a 440 × 440 × 644 image matrix with a voxel size of 1.65 × 1.65 × 1.65 mm3. A Gaussian post-reconstruction filter with 2-mm full width at half maximum (FWHM) was applied to the images as previously published [15].

CT scans were performed with equivalent parameters with slice thickness of 1.0 mm, pitch factor 1, bone and soft tissue reconstruction kernels, and maximum of 120 kV and 90 mAs by applying CARE kV and CARE Dose as previously published [1, 15].

### Image evaluation

Two nuclear medicine physicians (CM, LW) performed image evaluation and identified relevant lesions. Lesion size was determined as the largest diameter on the co-registered CT. For image evaluation, appropriate workstation and software were used (Syngo.via, Siemens Healthineers, Erlangen, Germany) [18]. Tumor lesion uptake was calculated using a 40% iso-contour volume-of-interest (VOI) approach [1, 19]. A 10-mm diameter spherical VOI in the right liver lobe characterized the background activity [20]. The VOIs were propagated to the corresponding lesions across all reconstructions to minimize misregistration.

### Semi-quantitative measurements

Background image noise was assessed by liver’s mean standardized uptake value (SUV_mean_) and standard deviation (SD). Signal-to-background ratio (SNR) was defined as the reciprocal coefficient of variation (σ = standard deviation of the background VOI/µ = background SUV_mean_). Tumor uptake was measured by maximum and peak standardized uptake values (SUV_max/peak_). This resulted in the calculation of the tumor-to-background ratio by SUV_max_ (TBR: SUV_max_(tumor)/SUV_mean_(liver)) and by SUV_peak_ (TBR_peak_ : SUV_peak_(tumor)/SUV_mean_(liver)). Image quality was analyzed by peak tumor-to-noise ratio (TNR_peak_) and a new image quality criterion (IQ).

$$\:TNR_{peak} =\frac{SUV_{peak}\left(tumor\right)}{SD\:\left(liver\right)}; IQ = TBR_{peak} \times TNR_{peak}$$

### Statistical evaluation

Statistical analyses were performed using Excel (Microsoft, Redmond, Washington) and Prism Version 10 (GraphPad Software Inc., San Diego, CA) [21]. Data are presented as mean ± standard deviation (SD). Comparisons between reconstructions in the same patient were analyzed using a paired two tailed Student’s t-test. P-values < 0.05 were considered statistically significant.

## Results

### Background noise on sSAFOV and LAFOV PET/CT

**Fig. 1:**
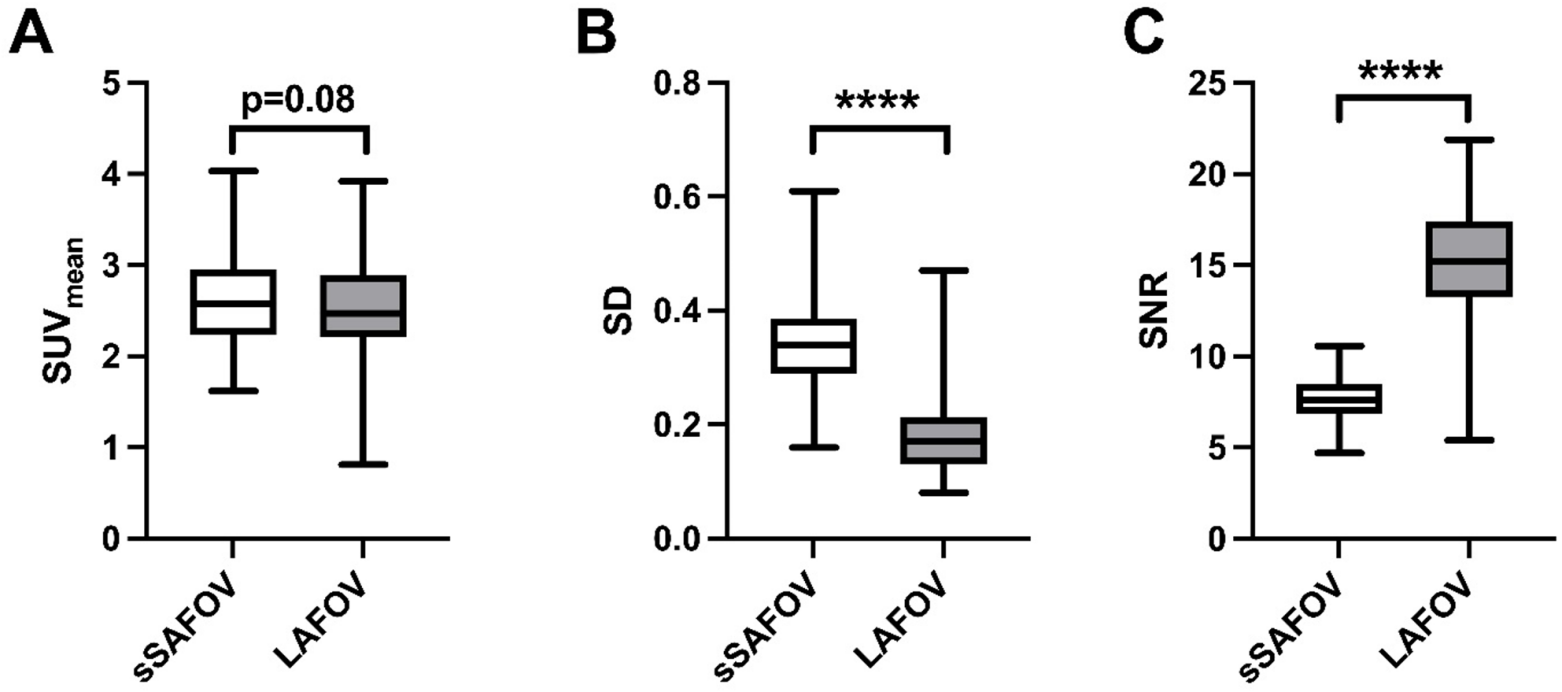
Background noise is characterized by liver’s mean standardized uptake value (SUV), standard deviation (SD) and signal-to-noise ratio (SNR). Compared are simulated short-axial field-of-view (sSAFOV) and long-axial field-of-view (LAFOV) reconstructions. Statistically significant differences are highlighted with an asterisk “*”

Background image noise was lower with LAFOV PET/CT reconstructions. While liver SUV_mean_ did not change significantly between both analyzed reconstructions (sSAFOV: 2.58±0.49 vs. LAFOV: 2.52±0.54, *p* = 0.08), SD was significantly higher in sSAFOV compared to LAFOV PET/CT (0.34±0.09 vs. 0.18±0.07, *p* < 0.0001). Subsequently, SNR was significantly lower in sSAFOV (sSAFOV: 7.71±1.22 vs. 14.98±3.81) Fig. 1.

### Tumor uptake by lesion size

In total, 160 malignant lesions were identified and categorized according to their largest diameter (< 8 mm: *n* = 22, 8–10 mm: *n* = 32, 11–20 mm: *n* = 48, 0.20 mm: *n* = 58) Table 1. [^18^F]FDG uptake in the malignant lesions differed between the analyzed reconstructions depending on the applied semi-quantitative measurement.

**Table 1:** Patient cohort includes melanoma, lung cancer, lymphoma, breast cancer, genitourinary cancer (GU), head and neck cancer (HNC) and Gastrointestinal cancer (GI)

Category	Subcategory	Sample size (*n*)
Included patients (*n* = 57)		
Tumor entity	Melanoma	20
	Lung cancer	17
	Lymphoma	6
	Breast cancer	2
	GU cancer	4
	HNC	4
	GI cancer	4
Age (years)		72 ± 12
Sex	Female	22
	Male	35
Analyzed lesions (*n* = 160)		
Lesion sizes (mm)	< 8	22
	8–10	32
	11–20	48
	> 20	58

Patients’ characteristics (mean±SD) indicate age, sex and number of malignant lesions per size

**Fig. 2:**
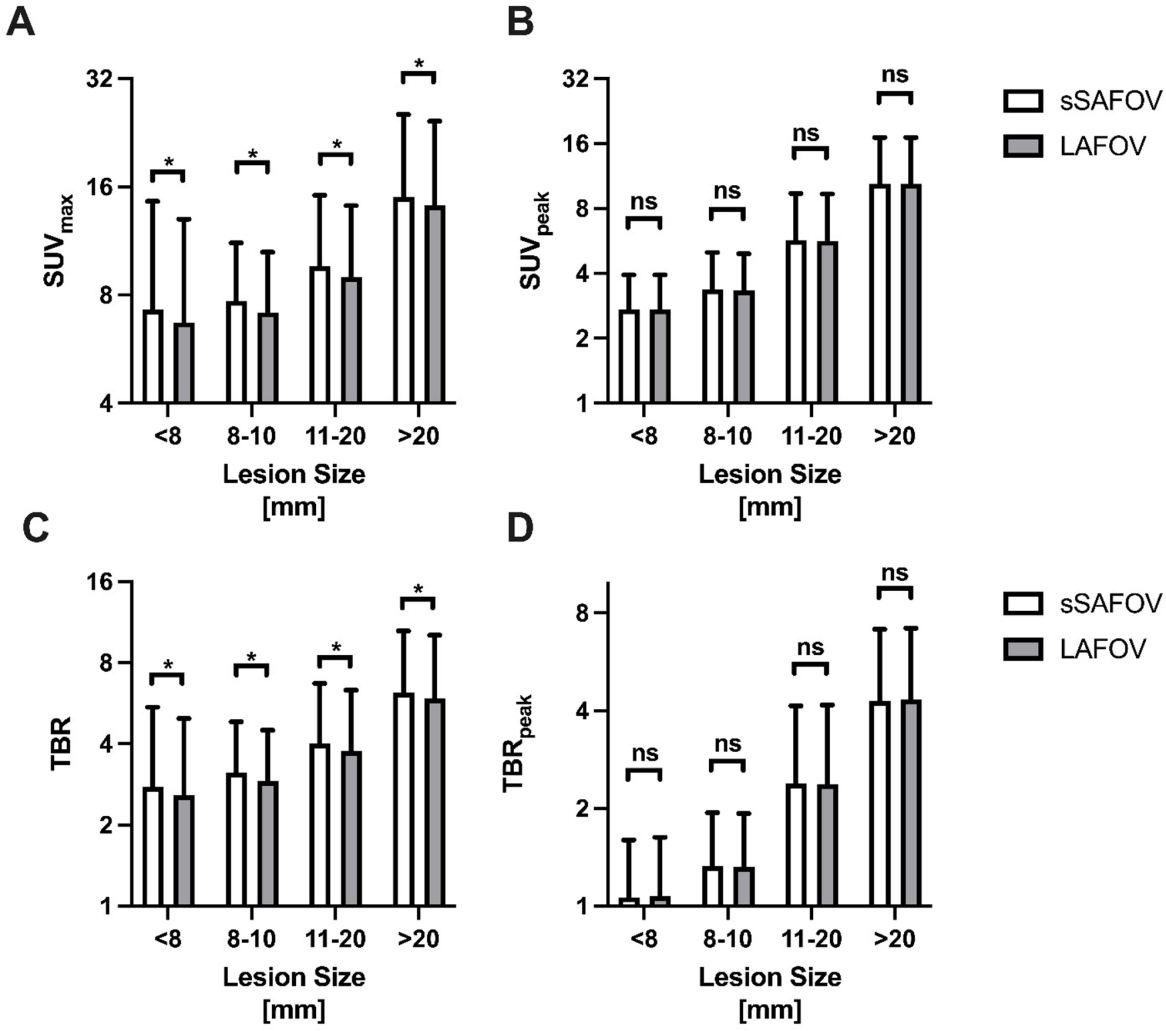
Tumor uptake is characterized by maximum/peak standardized uptake value (**A**: SUV_max_, **B**: SUV_peak_) and tumor-to-background ratio (TBR) of SUV_max_ (**C**) and SUV_peak_ (**D**). Compared are simulated short-axial field-of-view (sSAFOV) and long-axial field-of-view (LAFOV) reconstructions. Statistically significant differences are highlighted with an asterisk “*”, ns: not statistically significant

SUV_max_ was significantly higher for sSAFOV PET/CT in all lesion sizes compared to LAFOV PET/CT (< 8 mm: 7.28±7.31 vs. 6.71±6.29 *p* = 0.03; 8–10 mm: 7.71±3.46 vs. 7.16±3.37 *p* = 0.001; 11–20 mm: 9.64±5.52 vs. 8.95±5.22 *p* = 0.0001 and > 20 mm: 14.98±10.46 vs. 14.22±10.15 *p* < 0.001). TBR calculated by SUV_max_ was significantly higher for sSAFOV to LAFOV PET/CT (2.76±2.71 vs. 2.58±2.39 *p* = 0.03; 8–10 mm: 3.13±1.70 vs. 2.91±1.59 *p* = 0.0004; 11–20 mm: 4.01±2.39 vs. 3.76±2.56 *p* < 0.0001 and > 20 mm: 6.18±4.32 vs. 5.90±4.20 *p* < 0.0001) Fig. 2.

However, SUV_peak_ did not differ significantly between the compared reconstructions (< 8 mm: 2.73±1.21 vs. 2.73±1.21 *p* = 0.95; 8–10 mm: 3.38±1.62 vs. 3.34±1.60 *p* = 0.052; 11–20 mm: 5.72±3.66 vs. 5.66±3.68 *p* = 0.08 and > 20 mm: 10.39±6.71 vs. 10.39±6.70 *p* = 0.89). Subsequently TBR by SUV_peak_ was not significantly different between sSAFOV and LAFOV reconstructions (< 8 mm: 1.06±0.53 vs. 1.07±0.56 *p* = 0.47; 8–10 mm: 1.33±0.61 vs. 1.32±0.61 *p* = 0.44; 11–20 mm: 2.39±1.76 vs. 2.38±1.78 *p* = 0.69 and > 20 mm: 4.29±2.84 vs. 4.32±2.86 *p* = 0.12) Fig. 2.

### Image quality in different lesion sizes

**Fig. 3:**
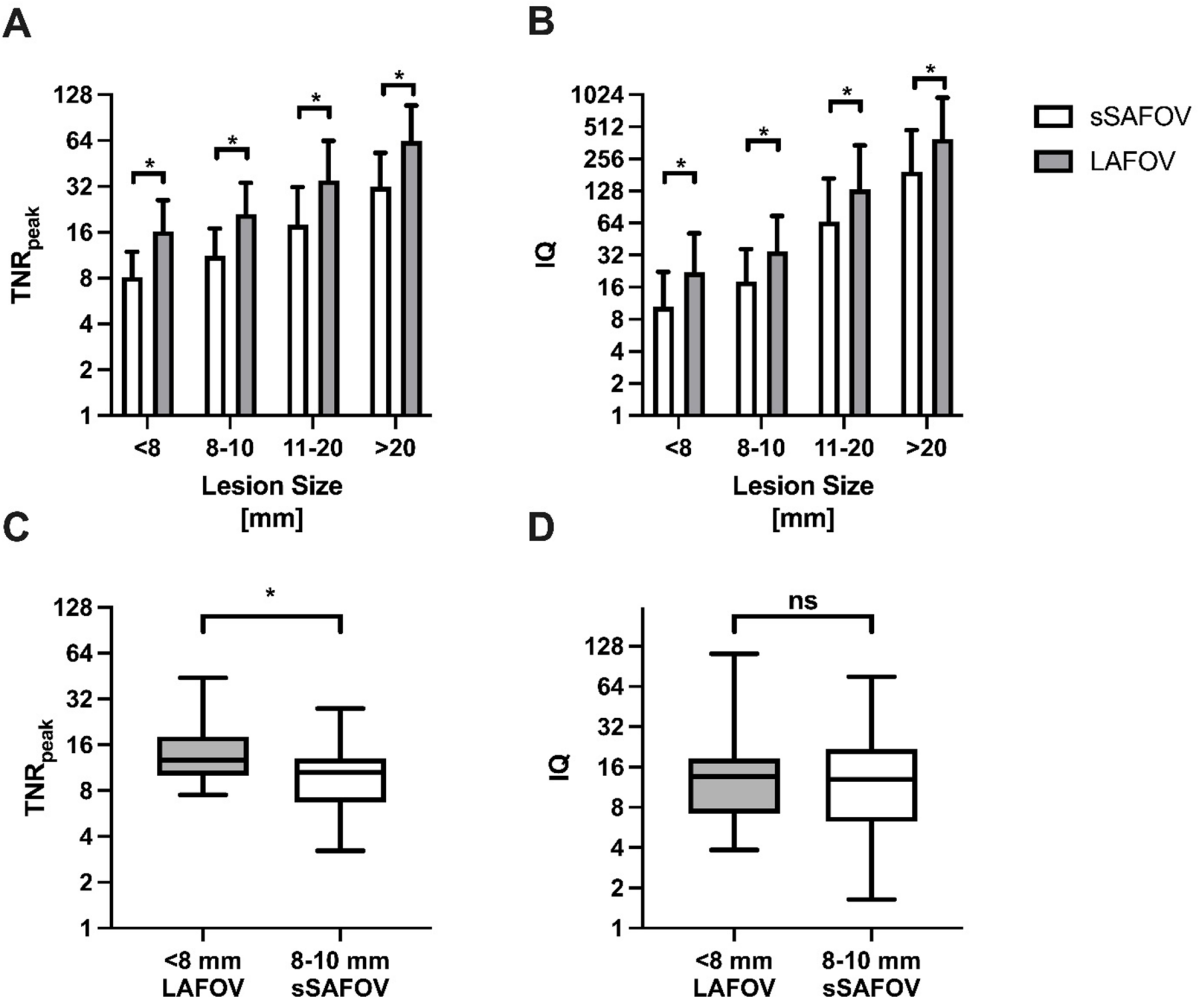
Image quality is characterized by tumor-to-noise ratio (TNR) of SUV_peak_ (**A**) and an image quality criterion (IQ, **B**). Compared are simulated short-axial field-of-view (sSAFOV) and long-axial field-of-view (LAFOV) reconstructions. TNR_peak_ between sSAFOV and LAFOV in the smallest lesion size (< 8 mm and 9–10 mm) is compared in **C** and corresponding IQ in **D**. Statistically significant differences are highlighted with an asterisk “*”

All LAFOV reconstructions exhibited higher image quality than sSAFOV PET/CT. TNR_peak_ was significantly higher in all analyzed subgroups (< 8 mm: 8.12±3.79 vs. 16.22±9.74 *p* < 0.0001; 8–10 mm: 11.18±5.70 vs. 21.09±12.67 *p* < 0.0001; 11–20 mm: 17.92±13.68 vs. 35.02±28.63 *p* < 0.0001 and > 20 mm: 31.85±21.26 vs. 63.78±45.01 *p* < 0.0001). Subsequently, IQ was significantly higher in LAFOV PET compared to sSAFOV PET (< 8 mm: 10.44±11.80 vs. 22.00±29.13 *p* < 0.01; 8–10 mm: 18.07±18.31 vs. 34.89±39.51 *p* = 0.0001; 11–20 mm: 66.04±101.66 vs. 131.76±210.50 *p* = 0.0001 and > 20 mm: 194.75±281.29 vs. 392.77±567.79 *p* < 0.0001) Fig. 3.

Of note, LAFOV’s TNR_peak_ in lesions < 8 mm surpassed of sSAFOV’s TNR_peak_ in lesions between 8 and 10 mm (< 8 mm LAFOV: 16.22±9.74 vs. 8–10 mm sSAFOV: 11.18±5.70 *p* = 0.02). There was no statistically significant difference in IQ in this comparison of the two smallest lesion size categories (< 8 mm LAFOV: 22.00±29.13 vs. 8–10 mm sSAFOV: 18.07±18.31 *p* = 0.54) Fig. 3.

## Discussion

We present first data evaluating the impact of LAFOV compared to sSAFOV PET/CT on cancerous lesions by size. We report significantly lower noise rates leading to significantly higher TNR in LAFOV PET/CT, especially for lesions < 8 mm.

In our study, liver’s SNR was significantly higher in LAFOV compared to sSAFOV (*p* < 0.0001) mainly due to significantly lower SD (*p* < 0.0001). This finding was in line to previous head-to-head comparative studies on LAFOV and TB PET/CT to SAFOV scanners [1, 22]. On a per lesion-basis we found significantly higher SUV_max_ values in sSAFOV compared to LAFOV PET, whereas SUV_peak_ did not show significant differences between both reconstructions Fig. 2. This finding is in line with previous rapports on decreased noise levels in SUV_max_, especially using the full acceptance angle (MRD 322) in the here used LAFOV system [9]. Subsequently, TBR was significantly lower on LAFOV compared to SAFOV PET in the same lesions in all lesion sizes (*p* < 0.05). Therefore, we introduced TBR_peak_, which did not show significant differences in both reconstruction. This finding is in line with previous studies suggesting that SUV_peak_ is a more reliable biomarker for lesion quantification [23].

Quantification indices according for differences in noise level (including SD_liver_) were significantly higher in LAFOV compared to SAFOV PET (*p* < 0.05) in all analyzed lesion sizes. Moreover, LAFOV PET/CT showed significantly higher TNR_peak_ in lesions < 8 mm compared to lesions between 8 and 10 mm with sSAFOV PET/CT. When combing quantification to background and noise level by our newly introduced image quality criterion (IQ: TBR_peak_ x TNR_peak_), IQ was significantly higher in LAFOV compared to sSAFOV PET/CT in all lesion sizes. In the two subgroups, LAFOV PET/CT showed comparable IQ for the smallest lesions (< 8 mm) compared to sSAFOV’s IQ for lesions between 8 and 10 mm (*p* = 0.54). LAFOV PET/CT showed higher lesion conspicuity in all lesion sizes compared to sSAFOV PET/CT. This was most useful for very small lesions (e.g. <8 mm), where LAFOV PET/CT outperforms sSAFOV, which might lead to higher diagnostic certainty in very small lesions. This new image quality criterion “IQ” was developed by our findings using UHS in LAFOV PET/CT [9]. We tried to combine the general background noise with both metrics, liver’s SUV_mean_ and SD as a reference organ for [^18^F]FDG to cancerous lesions’ uptake (SUV_peak_) to quantify readers’ image impression with regard to noise level. This metric is also transferable to other tracers and might be easily implemented into other centers’ protocols. We believe that the proposed IQ metrics can serve as an additional biomarker for PET image quality assessment, complementary to SNR and other parameters such as volume [24].

Some limitations need to be addressed. This retrospective evaluation focuses on a sSAFOV to LAFOV PET comparison. Only simulated SAFOV PET data were used like previously published [15]. Preferably, randomized prospectively enrolled cohorts could validate our findings. However, this study design allowed us to perform an intra-individual comparison, which helped mitigate potential biases. Moreover, we noted that melanoma and lung cancer lesions were overrepresented and the majority of lesions was ≥ 10 mm. Therefore, our study results might only be of limited transferability to other cancer lesions. Lastly, our finding is only applicable to one specific LAFOV scanner. We note an increasing number of different scanners designed by different vendors with different scanner characteristics. However, as the first LAFOV PET center with now two Biograph Vision Quadra systems, our data are of innovative nature.

## Conclusion

In this study, LAFOV PET/CT showed lower noise rates and higher tumor to noise ratios compared to sSAFOV PET/CT. SUV_peak_ was a more reliable biomarker compared to SUV_max_ for cancerous lesions. Smaller lesions’ quantification (< 8 mm) was impacted positively by next-generation LAFOV PET/CT. Image quality was significantly higher in LAFOV compared to sSAFOV PET/CT, which might positively affect diagnostic accuracy indices and diagnostic certainty in especially small cancerous lesions.

## Data Availability

The data are available upon reasonable request at the corresponding author’s address.

## Abbreviations

LAFOV: Long-axial field-of-view
TB: Total-Body
PET/CT: Positron emission tomography/computed tomography
SAFOV: Short-axial field-of-view
sSAFOV: Simulated SAFOV
NSCLC: Non-small cell lung cancer
MRD: Maximum ring difference
PSF: Point-spread-function
FWHM: Full width half maximum
SUV: Standardized uptake value
TBR: Tumor-to-background ratio
TNR: Tumor-to-noise ratio
IQ: Image quality criterion
GU: Genitourinary cancer
HNC: Head and neck cancer
GI: Gastrointestinal cancer
